# The Inflammability of Flannelette: An *Ex Parte* Statement

**Published:** 1916-10-07

**Authors:** Walter Street

**Affiliations:** Secretary, The Flannelette Association, 6 Major Street, Manchester.


*To the Editor of* THE HOSPITAL.

### The Inflammability of Flannelette: an Ex Parte Statement.

*To the Editor of* THE HOSPITAL.

SIR,—Your issue of September 23 contained an article dealing with the excellent work being done in connection with the operations of the Mothers' Institute, Johanna Street, Lambeth. Amidst a record of much excellent work, there appears one paragraph which is open to very strong exception. We are told that "flannelette is condemned at the institution, even though it cannot be altogether banished."

Why should the babies be deprived of garments made from the softest and most comfortable fabric there is, for which there would be substituted harder, rougher, and colder materials, peculiarly unsuited to the delicate skins of the little ones? In all probability the reason is that those who are responsible for this condemnation of flannelette imagine that there is greater risk of a burning accident occurring to one wearing that fabric than to those wearing alternative ones. That there is no ground whatever for this belief is well known to those who are best aware of the relative properties of flannelette and of other cotton fabrics which are used for similar purposes. No one who is acquainted with this subject would deny the advantages to be gained by attiring young children in under-garments or night attire made of good, soft, all-wool flannel; but, unfortunately, the cost of such fabrics places them altogether beyond the means of any except the very well-to-do classes. Even if all-wool materials were not practically prohibitive in price, the world's production of wool is so limited that it would only supply a small proportion of the population with garments, hence it follows that suitable substitutes, made from cotton, have to be found. Careful tests have shown that fabrics described as "woollen" frequently do not contain more than from 25 to 50 per cent. of wool, and that they are more inflammable than flannelette, costing half the price. Cotton materials, other than flannelette, costing about the same price, are quite as inflammable as that fabric. This is a fact with which the majority of people are unacquainted, but it has been established over and over again by scientific and impartial tests.

We have the strong testimony of a distinguished scientist, quite unbiassed and unprejudiced, to this effect. Professor William Thompson, F.R.S., F.I.C., etc., in the course of evidence he gave before a House of Commons Committee on "Coroners and their Duties," gave particulars of a number of experiments he had made to test this point, and stated that the result was that he had found there was no difference as to inflammability between flannelette and other cotton fabrics. He declared emphatically that in any of the suggested cases of death from burning, where the victims were wearing flannelette garments, exactly the same result would have arisen had they been wearing calico. On a recent occasion he also stated that "there is no more danger of fire from flannelette than from any other cotton fabric which has not been 'raised' and thus converted into flannelette."

It is a fact that flannelette has greatly benefited the public, being warm, soft, hygienic, durable, attractive in appearance, and in various ways more suitable for underwear and night attire than any other material to be obtained at equally low prices. It is unquestionable that during the last quarter of a century millions of persons have been warmly clad in this material who, but for its advent, would have been wearing inferior fabrics, many of them more costly and less durable, which would have involved them in a considerable amount of suffering and bad health.

Without claiming that flannelette has been the cause of the reduced death-rate, we may justly attribute a portion of the improvement to its beneficial effects.—Yours faithfully,

Walter Street, Secretary,
The Flannelette Association, 6 Major Street,
Manchester.

October 2, 1916.

[We print this letter to satisfy our correspondents, the Flannelette Association, who feel aggrieved by what has appeared in The Hospital on the subject of flannelette. We quite admit that there are two sides to this question, that many flannels are highly inflammable, and that even the best flannel will burn if given a favourable opportunity. Finally, we would refer our correspondents to the British Fire Prevention Committee, from whom we have received, within a few hours of the letter printed above, a set of simple fire precautions to be inculcated in institutions (for the blind). The sixth of these injunctions ends: "Do not use celluloid or flannelette in connection with your wearing apparel, as a mishap with an open light or fire may result in very serious burns if either of these materials becomes ignited . . ." It would seem that at the offices of the Fire Prevention Committee there exists a field for the Flannelette Association's missionary enterprise.—Ed. The Hospital.]

